# In-situ consolidation of thermoplastic composites by automated fiber placement: Characterization of defects

**DOI:** 10.1177/08927057241251837

**Published:** 2024-05-08

**Authors:** Mahmoud Fereidouni, Suong Van Hoa

**Affiliations:** 1 Concordia Center for Composites, Department of Mechanical, Industrial and Aerospace Engineering, 5618 Concordia University, Montreal, Quebec, Canada; 2 Research Center for High-Performance Polymer and Composite System (CREPEC), Montreal, Quebec, Canada

**Keywords:** Automated fiber placement, in-situ consolidation, thermoplastic composites, defects

## Abstract

The emergence of automated manufacturing of composites has not only transformed the manufacturing of optimized and geometrically complex structures but has also expanded the promising prospect of in-situ manufacturing of thermoplastic composites (TPC), where both material placement and consolidation are carried out by automated fiber placement (AFP) equipment, streamlining the process into single step manufacturing. However, the inherent complexities in different aspects of robotic automation, imperfections in the supplied material, and the occurrence of multi-physical phenomena during in-situ consolidation introduce various manufacturing-induced defects. While the defects in thermoset composites (TSC) made by AFP have been widely studied in the past, this study explores the diverse defects at micro and macro scales for TPCs made by AFP, with a focus on carbon-fiber/poly-ether-ether-ketone (CF/PEEK) tapes consolidated using hot gas torch (HGT) heating system. An overview of defects and associated characteristics is presented across three phases: defects in supplied impregnated tapes, defects and limitations in performance of AFP system, and defects in the final in-situ consolidated composite. For the defects subject to studies in the past, the description is limited to a concise review, while those with limited understanding are supported by new empirical observations in this work.

## Introduction

Although thermoset composites (TSC) have traditionally been the primary choice of manufacturers, there has been a steady upward trend in the adoption of thermoplastic composites (TPC) over the past three decades.^1^ Thermoplastics have gathered recognition for their superiority over thermoset counterparts in terms of manufacturing flexibility (i.e., weldability, repairability, thermoformability, short processing times, and infinite shelf life), mechanical properties (i.e. impact resistance, fracture toughness, fatigue life, high service temperatures), and environmental impacts (i.e. low fire, smoke and toxicity values).^1–3^ The emergence of automated manufacturing of composites marked notable progress in repeatability, productivity, and accuracy for producing large structures, while minimizing material scrap and labor costs.^4^ Automated Tape Laying (ATL) has been developed since the 1970s for high layup speeds with placement of wide prepreg tapes. Automated Fiber Placement (AFP) arose to incorporate capabilities of both tape placement and filament winding, enabling the manufacturing of complex or highly contoured open or closed section structures through the utilization of narrow prepreg tapes.^5–7^ AFP equipment, whether employing a robotic-arm configuration or a gantry setup, has a placement head that can support the deposition of either thermoset prepregs, thermoplastic prepregs, or dry fibers.

Excellent capacities and flexibilities of AFP equipment are offered at the expense of high inherent complexities, that inevitably lead to imperfections in different aspects of manufacturing, thereby introducing defects in the final product. Harik *et al.*^8^ introduced defect identity cards for a range of defect categories found in thermoset stacks made by AFP. They identified and classified defects into gaps/overlaps, pucker, wrinkle, bridging, imperfect boundary coverage, angle deviation, fold, twisted tow, wandering tow, loose tow, missing tow, splice, position error, and foreign object debris. Aside from the defect identification stage, the effects of AFP-induced defects on mechanical behavior of post-cured laminates have been investigated in several works, but were mostly limited to gap/overlaps. In various experimental studies on TSCs,^9–21^ gaps and overlaps with different sizes or patterns were introduced in laminates, and mechanical properties were assessed by different tests, namely unnotched tension/compression, open-hole tension/compression, in-plane shear, fatigue tests, and impact response.

While a large scope of research papers on AFP of TSCs is discernible, the availability of noticeably fewer publications on AFP of TPCs has been remarked,^22^ especially in terms of defect identification and the effects on mechanical response. The necessity for a separate route to advance research on defects in TPCs originates from conspicuous distinction between AFP-manufactured TSCs and TPCs in three aspects: in-process flow kinetics (percolation flow in thermoset cure process vs squeeze flow in thermoplastic in-situ consolidation), hardening mechanisms (chemical crosslinking vs physical solidification), and post-process material properties (modulus, ductility, fracture toughness). While certain defects in TPCs and TSCs can share identical names, they can be significantly different in terms of origin, formation mechanisms, geometrical/physical characteristics, and impact on mechanical performance. Even though plenty of work has been performed on the assessment of factors influencing the manufacturing quality of in-situ consolidated TPCs,^23^ a common focus was directed toward process modeling or process parameter optimization, rather than a comprehensive defect identification and characterization. Different mechanical tests, namely short beam strength, single lap shear, and mode I fracture toughness, along with measurement of void content and crystallinity content were implemented to assess the quality of in-situ made thermoplastics for various compaction forces, heat inputs, deposition speeds, and mandrel temperatures.^24–36^ Voids have been the only defect that was extensively studied for in-situ consolidation, and an acceptable insight into its effects on mechanical properties has been achieved.

Several useful review papers have been published to cover contributions in the domain of ATL/AFP, which can be found in the references 4,22,23,37–41. These reviews can be broadly classified into two categories. In the first category, the papers^4,22,37,38^ offer reviews of AFP defects (some of which include defect detection techniques or impacts of defects on mechanical properties), but do not intend to focus on TPCs, and predominantly revolve around defects in TSCs. The second category includes papers^23,39–41^ that review publications on AFP of TPCs, but are mainly focused on process modeling and process optimization efforts in the past, and do not aim to provide a complete picture of various defects and their characteristics. Therefore, there is a discernible need for clear-cut and thorough picture specific to defects in TPCs made using AFP. This paper does not aim to only provide a review on pertinent studies from the past but intends to present a research-based overview of defects and corresponding characteristics in three distinct stages, i.e., defects in supplied impregnated tape, defects and limitations in the performance of AFP system, and defects in the final in-situ consolidated composite. The defects in the final in-situ consolidated composite are further categorized into micro-scale and macro-scale groups. In each section, our findings and observations are accompanied by a concise description of relevant research contributions in the past. It should be noted that the findings in this work are based on observations in carbon-fiber/poly-ether-ether-ketone (CF/PEEK) tapes consolidated using hot gas torch (HGT) heating system. The utilization of other material systems or material suppliers, other heating systems (e.g., diode lasers), or other compaction rollers (e.g., soft silicone rollers) may result in different levels of intensity and frequency for the enumerated defects.

## Defects in supplied impregnated tapes

Some defects in the final thermoplastic composite part made by AFP may directly originate from imperfections in input material, rather than unoptimized process parameters or placement head flaws. In this section, these imperfections will be discussed in detail for commercial AS4/PEEK composite tape, which is extensively used for automated fiber placement of thermoplastics.

### Voids

**Figure 1.**
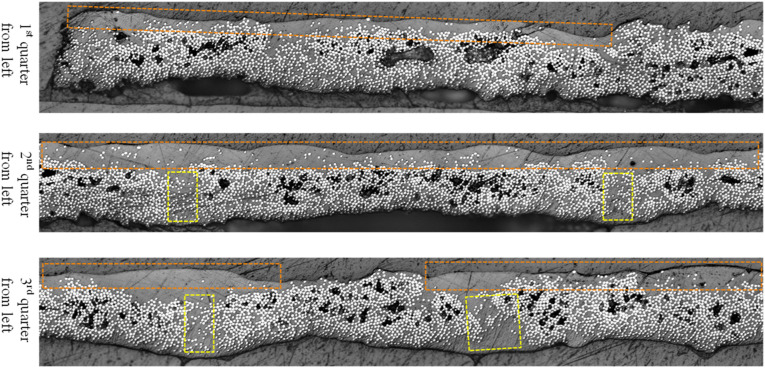
Cross-sectional view of Cytec APC-2 0.25-inch-wide impregnated tape.

Thermoplastic composite tapes and sheets intended for the production of high-performance parts exhibit relatively large initial void content, ranging from 5% to 10%.^42^ Microscopic cross section of a Cytec APC-2 tape is illustrated in Figure 1, where voids are nonuniformly distributed across the width with different sizes. Levels of void content within composites are inversely correlated with fatigue life, interlaminar shear strength, and flexural strength.^24,43,44^ Hence, void consolidation as a pivotal responsibility of AFP process is delegated to pressure applied via a compaction roller.

**Figure 2.**
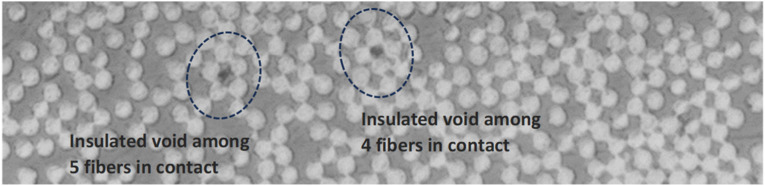
Insulated voids among densely packed fibers in thermoplastic composite.

Collapsing voids could be prevented by fiber-rich areas, in which interacting fibers bear the majority of pressure, leaving voids un-compressed.^45^ Figure 2 demonstrates trapped voids surrounded by touching fibers that hinder further contraction of voids. This feature highlights the significance of void content in raw tapes on final laminate defects, regardless of AFP process parameters themselves.

**Figure 3.**
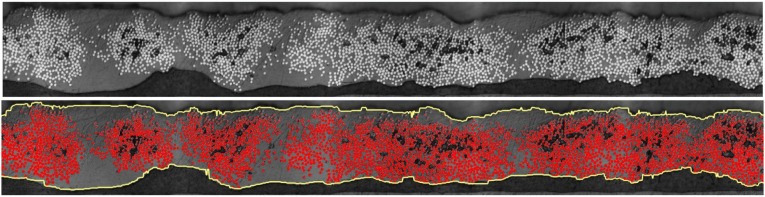
Edge and fiber detection in cross-sectional micrograph of Cytec APC-2 tape used for AFP (Top: input microscopic image, Bottom: output of image analysis by MATLAB program).

In the next two sections, the variation of fiber volume fraction and variation of thickness in supplied thermoplastic composite tape as additional types of imperfections will be discussed. A MATLAB code has been developed to perform image processing of micrographs in order to measure the variation of local thickness of tape and variation of fiber volume fraction along the width. The top and bottom edges of tape’s cross-section are detected using the gradient-based method by finding pixels with the maximum magnitude of the first derivative of RGB value. The fiber area was also recognized using prescribed threshold for RGB value. In Figure 3, the detected edges and fibers are displayed in yellow curves and red spots, respectively, for a sample of Cytec APC-2 impregnated tape used for AFP.

### Surface roughness and thickness variation

**Figure 4.**
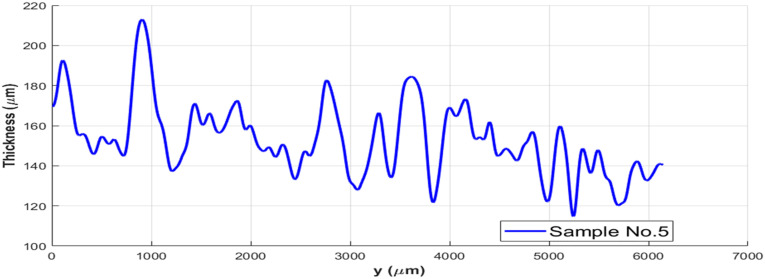
Variation of thickness along width of Cytec APC-2 impregnated tape used for AFP.

Commercially available thermoplastic prepregs have variations in the surface morphology and local thickness. In one study, a surface profilometry scan on Cytec AS4/PEEK tape showed an average roughness amplitude of 35 µm,^46^ which is a significant value compared with the average thickness of about 150 µm. From a different point of view, local thicknesses of 5 samples of 0.25″-wide Cytec AS4/PEEK were measured across the width using the image processing program and one is shown in Figure 4. The standard deviation of thickness across width for the studied samples is equal to 18.76 µm, accounting for 12.7% of average thickness. These morphological bumps must be squeezed so that surfaces of incoming tape and substrate come into full contact.^47^ Large levels of widthwise thickness variation on supplied impregnated tape may restrict development of intimate contact and create interlaminar voids.

**Figure 5.**
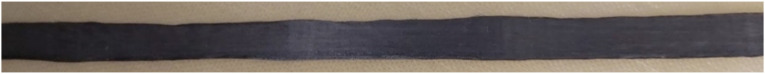
A band of APC-2 tape processed by automated fiber placement equipment.

However, the variation of width along the length of composite tape was observed to be insignificant using micrography of various samples, falling within the ±0.4% deviation from nominal width. Nonetheless, the variation of tape’s cross-sectional area along the length fell in ±4.5% range of deviation from the average value. Variation of cross-sectional area along length (or in other words, variation of average thickness along length given the minor width variation in supplied tape) may bring about elevated variation on local width of AFP-processed tape. Since the transverse flow of tape under the compaction roller is dependent on the initial thickness of tape, the post-processed local width of tape may appear non-uniform. Figure 5 illustrates the top view of a single AFP-processed APC-2 tape where variation in width is clear. This feature can potentially cause local gaps or overlaps when bands are parallelly placed to form a ply of laminate.

### Fiber content variation

**Figure 6.**
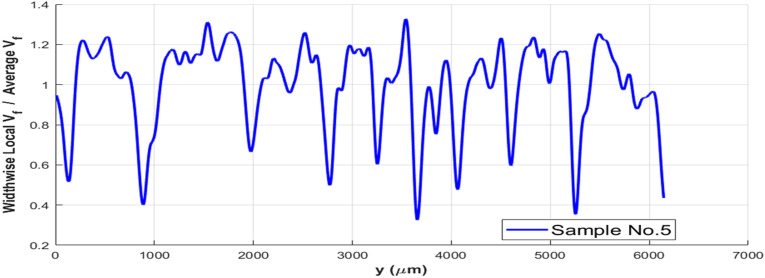
Variation of relative fiber volume fraction along width of cytec APC-2 tape used for AFP.

The variation of local fiber volume fraction across width of a Cytec AS4/PEEK 0.25″-wide sample is illustrated in Figure 6 for one of the five samples, measured by the image processing program. The cross-sectional images were discretized into 4419 elements along the width, and the fiber volume fraction was calculated for each element. The standard deviation of local fiber volume fraction across width is equal to 23.2% of the average fiber volume fraction.

Large resin-rich regions in thermoplastic composite prepregs could be categorized into two classifications; resin-rich *pockets* and resin rich *layer*. In Figure 1, four resin-rich pockets through the width of tape are marked by yellow dashed boxes. Moreover, resin-rich layers are shown by orange dashed boundaries, being located on the upper surface of the tape. Resin-rich *pockets* proved to reduce both transverse stiffness and failure strain of composites.^48^ On the other hand, thick resin-rich *layer* will create an area lacking fiber reinforcement between the plies, which can increase susceptibility to interlaminar failure in case of impact loading.^49^ For in-situ consolidation of thermoplastic composites, however, a thin resin-rich layer can improve the welding of incoming tape to the substrate. Accudyne Systems manufactured various samples using AFP in which impregnated tapes were devoid of surface resin-rich layer. The resulting laminates showed a significant number of interlaminar voids.^45^ It is proposed that a surface resin layer with a thickness equal to one filament diameter is sufficient for complete layer-to-layer weld development.^50^

### Fiber angle variation

The maximum axial load-bearing capacity of reinforcing fibers is attained when they are oriented in straight lines along the applied load path. However, the wavy nature of free individual filaments plus transverse loads applied via neighboring fibers induce deviation in local fiber angle from their desired value. Hsiao and Daniel^51^ analytically and experimentally showed that increasing fiber waviness significantly diminishes both Young’s modulus and compressive strength of unidirectional laminates. It was found out that locally misaligned fibers create local shear stress which could be significant enough to initiate interlaminar shear failure and delamination. Considering the inferior interlaminar shear strength of in-situ consolidated thermoplastic laminates to that of autoclave consolidated counterparts,^52^ the fiber waviness can even bring about a more critical impact on the failure of thermoplastic composites made by automated fiber placement.

**Figure 7.**
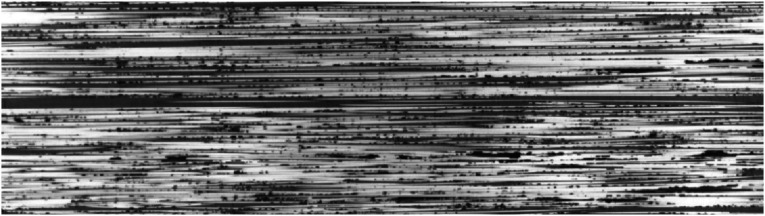
Microscopic top view of fibers in APC-2 tape used for AFP (area of sample covered in the image: 4.5 mm × 1.3 mm).

**Figure 8.**
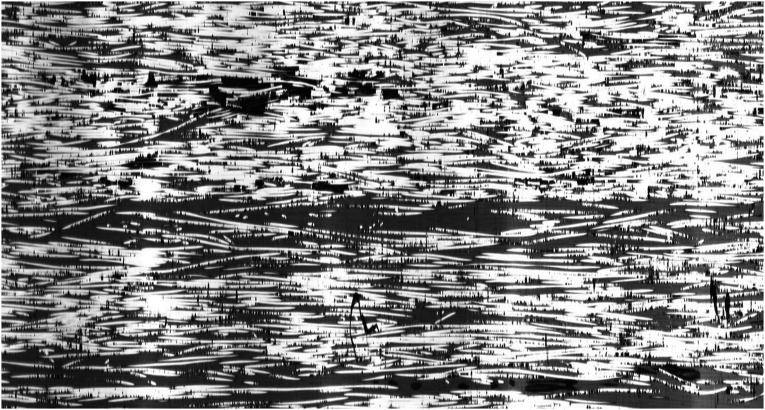
Vertically stretched microscopic top view of fibers in APC-2 tape used for AFP (area of sample covered in the image: 13.2 mm × 0.7 mm).

A prerequisite stage to analyze fiber waviness in thermoplastic composites made by automated fiber placement is to gain an insight into the magnitude of initial fiber waviness in supplied tapes before the process. A Cytec APC-2 CF/PEEK tape was polished from the top surface for micrographic observation. The plane of cross-section is parallel to the plane of tape, showing the top view of fibers running along longitudinal direction of tape. A small part of area sized at 4.5 mm × 1.3 mm is presented in Figure 7. Although individual filaments are observable as white horizontal lines, direct measurement of in-plane fiber waviness is not possible due to the small amplitude of waviness and discontinuity of fibers in the cross-sectional view (most fibers fully cross the micrographic cutting plane at some point, rather than remaining in cutting plane through a long path). An additional representation of fibers in the same tape, but spanning a larger length of the tow, is presented in Figure 8. The micrograph shows an area measuring 13.2 mm in length and 0.7 mm in width, while the image is vertically stretched by a factor of 10 to increase the visibility of waviness. In the next step, a method for calculating estimated fiber waviness based on fiber angle deviation is presented.

**Figure 9.**
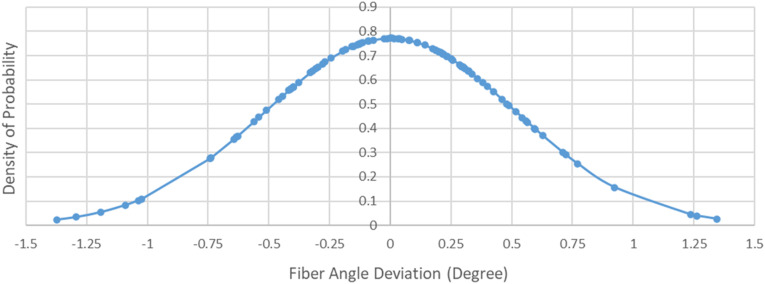
Normal distribution of in-plane fiber angle misalignment in APC-2 tape.

On a larger micrographic image covering an area around 20 mm in length and 3 mm in width, one hundred fibers were selected randomly out of hundreds of observable fibers. Micrograph was imported and analyzed in SolidWorks (Software by Dassault Systems), where short line segments were fitted to fibers, and angles were recorded. Particularly, the exact angle of each selected fiber with respect to the horizontal axis of the micrograph was measured. The angles varied from −0.97° to 1.74°, with mean value of 0.401°. To factor out the off-axis angle of the micrograph image, the mean value was subtracted from fiber angle values. Therefore, fiber angles with respect to the mean value (principal axis of tape or ideal orientation of fiber) varied within range of −1.37° to 1.34°. The standard deviation of values was calculated to be 0.517°, and 71 out of 100 measured angles fell within the first standard deviation. The normal distribution of values is presented in Figure 9.

**Figure 10.**
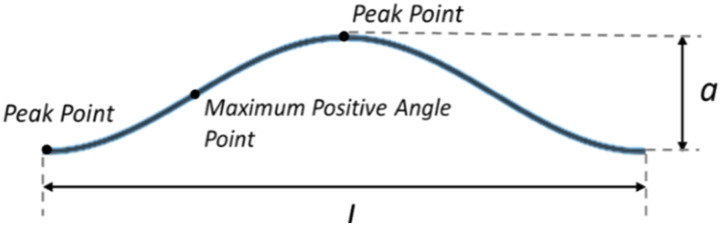
Representation of fiber path as a sine wave.

If fibers follow sinusoidal wave trajectory (Figure 10), then the transverse deflection can be^53^:

(1) $$y=\frac{a}{2}\left[1-\;\cos\;\left(\frac{2\pi x}{L}\right)\right]$$

Where a is peak to peak distance and L is the wavelength. The deflection angle is:

(2) $$\theta =\frac{dy}{dx}=\frac{\pi a}{L}\;\sin\left(\frac{2\pi x}{L}\right)$$

The maximum positive deflection angle occurs at x=L/4 where:

(3) $$\theta_{\max}=\frac{\pi a}{L}=\frac{\pi }{\beta }$$

Where β=L/a is waviness ratio^53^ and $\theta_{\max}$ is in Radians. The maximum positive angle in degrees simply is:

(4) $$\theta_{\max}=\frac{180}{\beta }$$

The peak points are located at x=zL/2 where z = {…,-2,-1,0,1,2,…}, on which fiber angles are apparently zero. In the meantime, points of maximum positive deflection angle are located at x=zL+L/4 . It can be deduced that the frequency of zero deflection angle is twice the frequency of maximum positive deflection angle. In other words, if one selects a random point on fiber trajectory, the possibility of taking a peak point (zero angle) is two times larger than the possibility of taking a maximum positive angle point. Meanwhile, the probability density function for the problem is:

(5) $$f\left(\theta \right)=\frac{1}{\sigma \sqrt{2\pi }}e^{-\frac{1}{2}{\left(\frac{\theta }{\sigma }\right)}^{2}}$$

where σ is the standard deviation. The probability density at θ = 0 is:

(6) $$f\left(\theta =0\right)=\frac{1}{\sigma \sqrt{2\pi }}$$

The probability for $\theta_{\max}$ would be a half of probability for θ=0 :

(7) $$f\left(\theta =\theta_{\max}\right)=\frac{1}{\sigma \sqrt{8\pi }}$$

On the other hand, in order to find out $\theta_{\max}$ , one can note:

(8) $$e^{-\frac{1}{2}{\left(\frac{\theta_{\max}}{\sigma }\right)}^{2}}=\frac{1}{2}\;\to \;-\frac{1}{2}{\left(\frac{\theta_{\max}}{\sigma }\right)}^{2}=\ln\frac{1}{2}\;\to \;\theta_{\max}=\sqrt{-2\;\ln\;0.5}\;\sigma$$

Substituting equation (8) into equation (4) results in:

(9) $$\beta =\frac{180}{\sqrt{-2\;\ln\;0.5}\;\sigma }=152.88\;\sigma^{-1}$$

**Figure 11.**
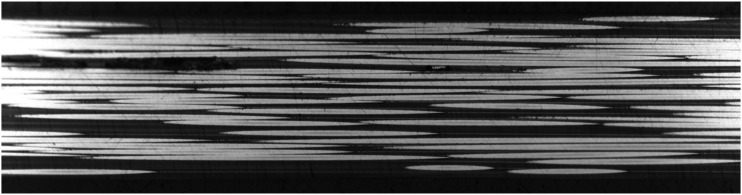
Microscopic side cross-section of APC-2 tape (cutting plane parallel to the fibers but perpendicular to the plane of tape).

Thus, the selected population of fiber angles, whose standard deviation is 0.517°, proposes the fiber waviness ratio of 296 for the APC-2 composite tape. This value represents the level of in-plane waviness of fibers, since the micrograph depicts the top view of the tape. A similar process has been carried out to measure the out-of-plane waviness of fibers in the tape. Figure 11 shows cross-sectional side view of the tape (cutting plane parallel with fibers but perpendicular to the plane of tape). The standard deviation in fiber angles was 0.504°, signifying an out-of-plane waviness ratio of 303. The values reveal that the severity of waviness is relatively minor in the thermoplastic prepregs, and moreover, the in-plane and out-of-plane waviness ratios are in close proximity.

While samples from one roll of Cytec APC-2 CF/PEEK tape were evaluated in this section to list defects of impregnated TPC tapes, it is essential to recognize that the levels of defects in different batches, or in other material systems, or products from different suppliers can vary significantly.

## Defects and limitations in performance of AFP equipment

Automated fiber placement machines can be characterized by their substantial size and complexities, mostly found in a gantry or robotic platforms. The complexities and imperfections in robotic components, composite placement head, and controller can result in deviations from the expected or nominal performance of equipment in manufacturing. The following categories explain the common imperfections in operation of equipment and resulting defects in thermoplastic composite material.

### Positioning error

Manufacturing of high-performance structures using AFP requires placing a large number of narrow strips of impregnated fibers. Deviation in following programmed trajectories leads to gaps or overlaps between adjacent strips. The positioning precision of AFP equipment can be subject to influence from kinematic errors and force-induced errors.^54^ Kinematic errors cause the robotic head to deviate from the desired path in motion, even if no load is applied on the head. They mainly stem from assembly flaws or geometric imperfections in robotic components, especially in joints. Besides, force-induced errors emerge when applied loads elastically deform components (e.g. links and joints), and consequently, displace the tip of placement head. The applied loads could be divided into gravity force due to weight of components and also compaction force during process. For large AFP machines the mass of links and placement head could create enough torque to deform joints significantly.^55^ In AFP of TPCs, compaction force is the crucial part of the process, where typically hundreds or sometimes thousands of newtons should be applied for acceptable consolidation. This concentrated force at the tip of the robotic arm may create enough torque on joints to deviate the nominal position of head and create gaps/overlaps and secondary defects in the laminate.

### Material feeding and cutting imperfections

The thermoplastic composite materials employed in the AFP process are provided in form of spools of slit tapes. One spool consists of pieces of slit tapes that are connected by their overlapping ends. The slit tapes can be usually cut from a larger unidirectional sheet with finite size, and then, the tapes are joined together on their ends using adhesive tape. The splice on the tape must be detected using a sensor during deposition of material. Once detected, the process is stopped, the splice must be cut and removed, and process restarts by reintroducing material. Occasional error in detecting splice results in implanting adhesive tape of splice into the composite laminate, which impairs local interlaminar bonding. On the other hand, numerous electronic and power subsystems are densely integrated in the AFP equipment. A signal between two subsystems may occasionally be missed due to electrical noise. When a signal is missed head events such as head extend, material feed and cut could potentially fail to occur during a part program. This is one of the reasons causing missing tape defect to take place in the process. Another observed fault is the discontinued material feeding due to finished material on spool, while the placement head continues to follow the course on the part with no deposition.

**Figure 12.**
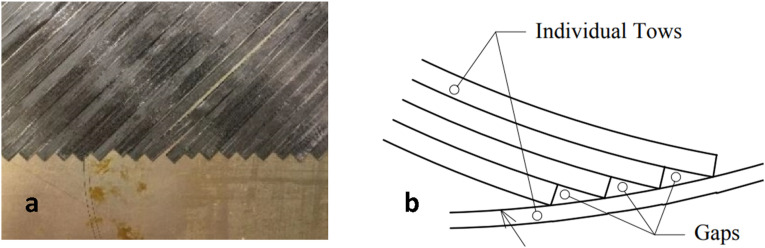
Boundary coverage in AFP (a) external boundary ^8^ (b) internal boundary^56^.

Current AFP machines use cutting systems in which tape is cut perpendicular to fibers. When orientation of fiber path is neither perpendicular nor parallel to the boundaries (e.g., creating a 45-degree ply), the coverage of boundaries could be defective (Figure 12(a)). For achieving the final desired shape, the unavoidable zigzag-shaped edges must extend beyond the intended straight boundaries to allow for the subsequent trimming of the excess edges. In thin laminated thermoset composites made by AFP, cutting away excess edges can be performed before curing cycle when material is soft, rather than after the solidification of resin. This can minimize the chance of introducing micro-cracks or delamination on the edges of final part. For the case of thermoplastic composites, however, cutting excess edges of final consolidated part is the only option, which may potentially impose points for crack initiation or delamination on edges. Furthermore, manufacturing of composite parts with complex geometries, customized lamination, or variable stiffness may involve a combination of depositing strips with different lengths and with different orientations within a ply. The start points and/or end points of some strips could be within a ply rather than on the external boundaries, which may create large triangular gaps or overlap areas (Figure 12(b)).

### Heating system limitations

Laser and HGT are considered the two most common heating systems employed for the in-situ consolidation of TPCs.^57^ While HGT heating systems offer low capital costs, low safety concerns, and design flexibility, they are accompanied by low energy efficiency, long response time, and weak controllability over the heated area.^40,58^ Diode laser heating systems, which have gained recent prominence, provide high heat flux and energy efficiency, fast response time, and focused heating.^59^ However, the main limitations of lasers include high initial purchase costs and the requirement for strict safety precautions.^60^ In addition, since the diode laser energy is primarily absorbed by carbon fibers, in the case of manufacturing glass fiber reinforced thermoplastics, the diode lasers lose their efficiency. In a different context, challenges in heating near nip-point area arise due to flow stagnation for HGT systems and radiation shadow for laser systems.^39,61^ The use of water-cooled silicone rollers on laser-assisted AFP systems brings about relatively fast cooling of incoming tape in the shadowed area before consolidation, which necessitates increasing tape temperature beyond typical processing temperatures in laser-exposed area.

## Defects in final in-situ consolidated composite

### Micro-scale defects

#### Gaps and overlaps

A substantial number of studies have been conducted on the characteristics of gaps and overlaps and their effects on the mechanical performance of thermoset composites made using AFP/ATL.^16,20,62–64^ Sawicki and Minguett^65^ revealed significant losses in compressive strength of laminates containing overlap or gaps. Woigk *et al.*^15^ showed that combination of gaps and overlaps gave rise to strength reductions in compression and tension by 14.7 % and 7.4%, respectively, whereas isolated gaps or overlaps did not bring about a major knockdown to strength. Fayazbakhsh *et al.*,^63^ however, reported that gaps diminish both buckling load and in-plane stiffness, while overlaps enhance structural performance. In almost all studies, AFP-stacked samples were made of Carbon-Fiber/Epoxy prepregs and underwent autoclave cure cycle. In situ-consolidation of thermoplastic composites, however, features clear differences in various aspects of processing parameters (e.g., pressure, temperature, time scale), in-process material properties (e.g., viscosity, permeability, solidification mechanism), and post-process material properties (e.g., modulus, ductility, toughness).

**Figure 13.**
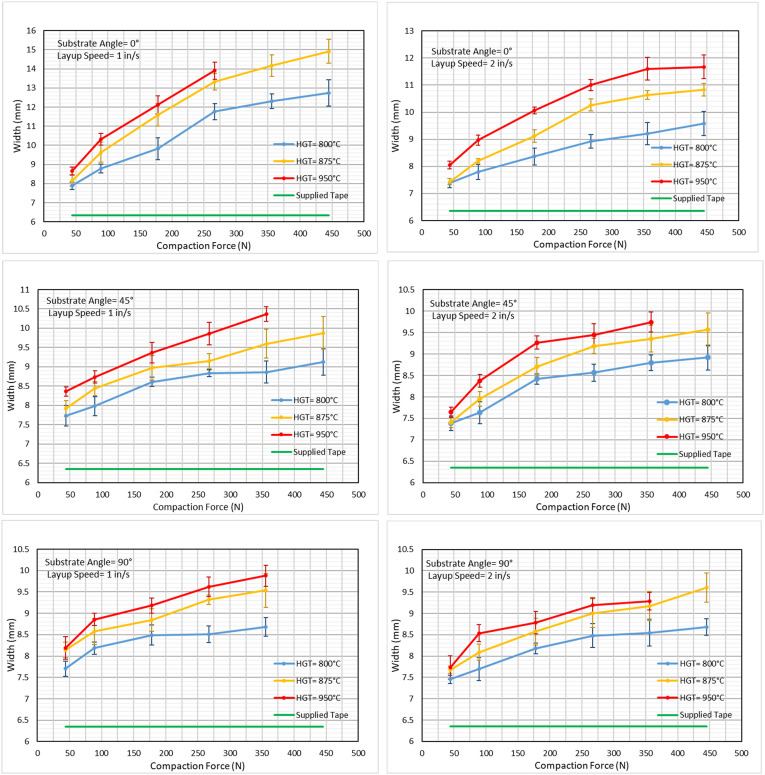
Widths of tapes in-situ consolidated using AFP with various process parameters and substrate fiber angle.

One major cause of gaps and overlaps in AFP involves placement head positioning errors. Additionally, in manufacturing of complex geometries using fiber steering, gaps or overlaps may inevitably appear.^8^ Clancy *et al.*^66^ showed that the final width of steered tape is directly correlated with steering radius, affecting the size of gaps or overlaps between neighboring tapes. Besides, particularly in AFP of TPCs, the comparatively large localized pressure on molten incoming tape gives rise to transverse flow of the tape, which increases width and reduces thickness. For example, if fiber paths and distance between adjacent courses are programmed based on width of supplied input tape, overlapping areas would be omnipresent in final laminate due to process-induced width increase. On the other hand, predicting the local width of consolidated tapes could be challenging since the amount of squeeze flow is dependent on various parameters including tape initial dimensions, nip point temperature, consolidation pressure/length/time, and tape-roller or tape-substrate friction. In an experimental evaluation, the widths of Cytec CF/PEEK tapes in-situ consolidated on composite substrate (i.e., CF/PEEK unidirectional sheet) have been measured for various combinations of process parameters and substrate fiber orientation. Three substrate fiber angles (0°, 45°, 90°) with respect to the axis of deposition, two layup speeds (1 in/s, 2 in/s), three hot gas torch temperatures (800°C, 875°C, 950°C), and six compaction forces (ranging from 10 lbf to 100 lbf) have been implemented to manufacture total of 108 single-layer samples using HGT-assisted AFP equipment at Concordia University. The results of measurements on final widths are presented in Figure 13. It should be noted that manufacturing certain samples was unfeasible due to excessively high temperatures or compaction forces. In such cases, the tapes typically wrapped around the roller rather than adhering to the substrate, resulting in a few missing data points in the figures.

As evidenced by empirical observation, an increase in torch temperature leads to a decrease in matrix viscosity, consequently resulting in larger widths. On the other hand, decreasing layup velocity prolongs the heating time of the incoming tape, leading to elevated tape temperatures and ultimately larger final widths. Moreover, escalating compaction forces impose higher squeeze flow and wider final widths, albeit with a non-linear relationship typically exhibiting concave-down behavior. Samples placed on a 0° substrate exhibit larger widths compared to those placed on 90° or 45° substrates. This phenomenon could be attributed to the 0° substrate experiencing simultaneous transverse squeezing without imposing significant transverse resistance or interfacial friction on the incoming tape. Samples on 90° or 45° substrates display marginal differences in width when other parameters remain constant.

AFP equipment utilizing soft rollers may impose smaller transverse deformation on APC tapes. In a study on the deformation of CF/PAEK tapes consolidated via soft roller on flashlamp-assisted AFP equipment,^67^ the range of increase in width fell between 5% and 13% by the variation of compaction force, nip-point temperature, and heated length over a normal scope.

**Figure 14.**
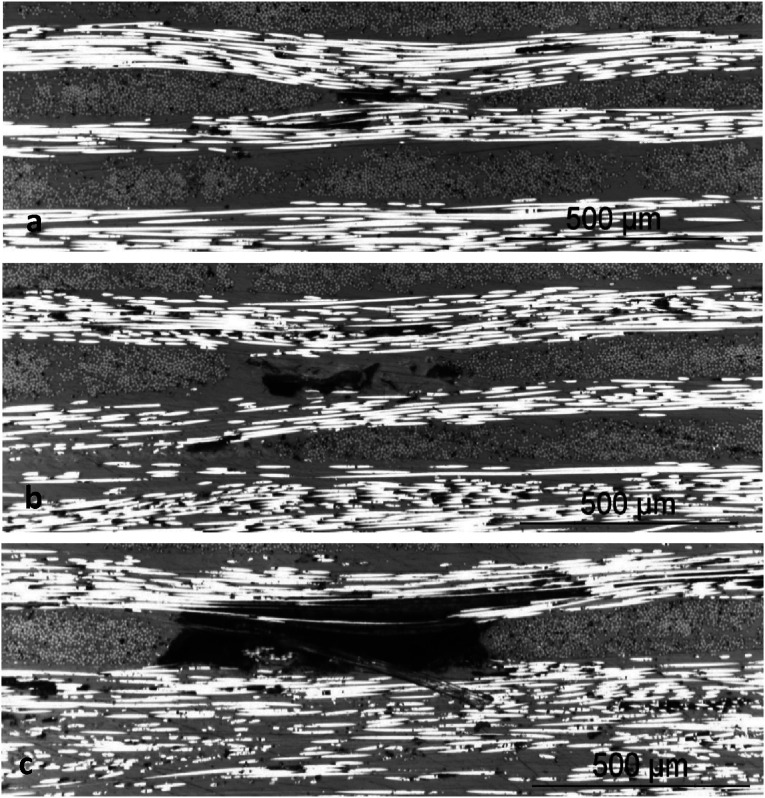
Gaps sized at (a)350 µm, (b)550 µm, (c)750 µm in cross-ply thermoplastic laminate made by AFP.

To identify micro-scale characteristics of gaps and overlaps in thermoplastic composites, a 12-layer cross-ply laminate including deliberately introduced gaps and overlaps with different sizes was manufactured using HGT-assisted AFP equipment. The values of process parameters used for HGT temperature, HGT flow rate, and compaction force were 875°C, 60 SLPM (Standard Liters Per Minute), and 40 lbf, respectively. The cross-sectional views of three gaps with different sizes (around 350 µm, 550 µm, and 750 µm in width, respectively) in the laminate are shown in micrographs of Figure 14. One common feature of gaps is the resulting empty areas (inter-band voids or holes), whose sizes are proportional to the width of gaps. This is in contrast with gaps in thermoset composites, where percolation flow of resin fills up the gaps, creating large resin pockets.^15,68^ The magnitude of viscosity for PEEK resin in processing temperatures outweighs that of Epoxy (in the initial stage of autoclave processing) by a factor of at least 1E3. Moreover, the time scale of in-situ consolidation is smaller than that of autoclave processing by a magnitude in range of 1E4 to 1E5. However, the in-situ consolidation pressure may exceed typical autoclave pressure used for thermoset prepregs by a range of 1E1 to 1E2. With reference to mentioned orders of magnitude and utilizing Darcy’s description, it could be estimated that percolated volume of resin in autoclave processing would be larger than in-situ consolidation by a scale of 1E6. Therefore, high viscosity and short time in AFP of TPC constrain the flow of resin into gaps, increasing the chance of leaving holes in composite. Although the percolation flow may still slightly contribute to filling gaps, it appears inefficient and limited for independent filling of large gaps during in-situ consolidation. The major controlling mechanism for filling gaps is squeeze (affine) flow. In Figure 14(b) as an example, the effective width filled by percolated flow is measured to be 337 µm (area of neat resin in gap divided by average thickness), while the measured increase in total width of tape (measured on the larger micrograph covering the whole width of consolidated tape) is equal to 3272 µm, highlighting the significance of squeeze flow in gap filling.

**Figure 15.**
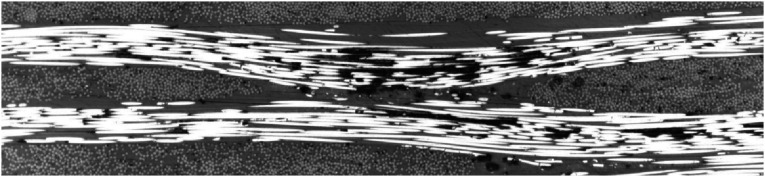
Gap-induced deconsolidation (evident by voids) in a ply placed on gap.

Gaps may also give rise to two secondary defects – i.e., deconsolidation and out-of-plane waviness in the ply placed on the gap. By deposition of a layer onto a layer including gaps, the compaction pressure on incoming tape vanishes locally on gap, where there is no/partial contact between incoming tape and substrate. A severe deconsolidation is evident in Figure 14(c), in which the lower half of the layer passing over gap fell apart due to zero pressure on the lower side. Figure 15 depicts a milder case of gap-induced deconsolidation, characterized by the presence of substantial amounts of void. The other secondary defect, gap-induced waviness, will be discussed in the next section.

**Figure 16.**
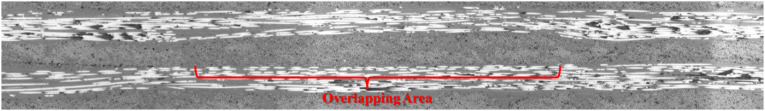
Overlapping bands in cross-ply laminate made by AFP.

**Figure 17.**
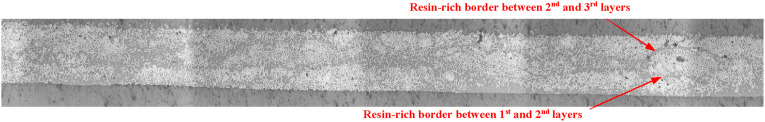
Unidirectional laminate made of three APC-2 bands using AFP.

The various gap-induced defects may bring about questions on overlaps as less severe counterparts. An overlap of two neighboring tapes, sized 1500 µm in width, is illustrated in Figure 16. After microscopic inspections at different points, no sign of elevation of voids was observed in the overlapping area of neighboring tapes in the laminate. In Figure 16, by a thorough observation, the boundary between overlapped tapes could be tracked down via thin interlayer resin-rich areas at some locations. Upon application of sufficient compaction pressure during AFP of TPC, the fibers of incoming tape partially penetrate into section of substrate (if incoming tape and substrate have the same fiber orientation). This creates bumpy interface and increases interaction between two layers. A unidirectional laminate made of three CF/PEEK bands with the same process parameters is shown in Figure 17. The bumpy boundary between intertwined layers is detectable at some of the areas. The increased interfacial area (for a constant projected area) has favorable effect on interlaminar bonding strength.^69^ The overlapping area as an example of squeezed same-angle strips, creates an intertwined and cohesive area. The utilization of tapes featuring a higher fiber volume fraction, reduced resin-rich areas, and a more uniform thickness can restrict the development of the mentioned effect for interfacial intermix of fibers. Nevertheless, the local thickness of ply at the overlap area of tapes is obviously larger than the regular areas. The average thickness of overlap area and average thickness of tapes on both sides were measured by image processing of Figure 16. The average thickness of processed tapes is 108 µm while the overlap area has average thickness of 126 µm, indicating an increase of 17% only. Unlike what simple speculation may suppose, the overlap area is not twice as thick as the regular area, based on observations. The pressure applied by roller is locally intensified at overlap area owing to elevated thickness in that region. Hence, the transverse flow at the overlap area is magnified when compared to the rest of ply. This phenomenon, that is more evident when a hard compaction roller is used, attenuates overlap-induced undulations and lessens the out-of-plane waviness imposed on the next plies. Soft compaction rollers, commonly employed in laser-assisted AFP systems, may not necessarily yield the same favorable outcome in flattening overlaps.

#### Out-of-plane waviness of plies/fibers

The out-of-plane waviness of plies in thermoset composite laminates and its impacts have been widely studied since 1994. Chun et al.^70^ have provided theoretical explanations and experimental evidence highlighting the remarkable impact of out-of-plane fiber waviness on the compressive and tensile elastic properties in graphite/epoxy unidirectional composites. In an experimental study by Wu C et al.^71^ on unidirectional laminates made of carbon-fiber/epoxy prepreg, the outcome of introducing out-of-plane wrinkle with 0.037 waviness severity  was reductions in compressive modulus and strength by 14.4% and 33%, respectively. They have also demonstrated that the sensitivity of compressive strength to out-of-plane waviness is greater than its sensitivity to in-plane undulations. Fewer studies have been carried out on the effect of waviness in TPCs. In a relevant work by Adams and Hyer^72^ on the influence of layer waviness on static performance of TPCs, reductions up to 36% in compression strength have been reported when out-of-plane waves (waviness severity ranging within 0.02 to 0.08) were introduced in the central layer of symmetric laminates made of carbon-fiber/polysulfone. Due to the significance of the defect, in this section, different mechanisms for development of out-of-plane waviness in TPC made using AFP are explained:

##### Gap/overlap-induced out-of-plane waviness

Gaps create weak or empty regions into which the next layer sinks, resulting in localized out-of-plane waviness. For narrow gaps, the length of descended area (wavelength) in the layer placed on gap surpasses the width of gap. The amplitude of wave (half of peak-to-peak height) is smaller than half of the consolidated ply thickness for narrow gaps (where the top ply cannot touch the bed of gap), and is equal to half of the consolidated ply thickness for wide gaps (where the top ply touches the bed of gap). The overlap-induced waviness proved to be less severe in terms of waviness ratio upon application of sufficient compaction pressure. Amplified transverse flow at overlap area can decrease amplitude of waviness for the next layer, resulting in an alleviated waviness ratio.

##### Out-of-plane waviness induced by resin/fiber-rich areas

**Figure 18.**
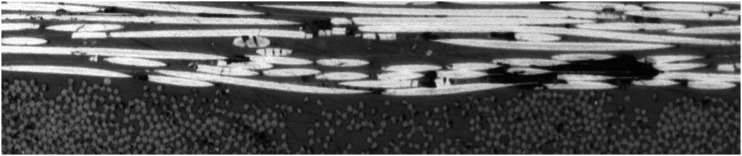
Out-of-plane waviness of fibers due to sinking in resin rich area of previous ply.

Once a ply is deposited, out-of-plane waviness may arise if large resin-rich pockets exist in a previous ply. During deposition, the molten substrate (as the foundation for incoming tape) is softer in resin-rich areas and harder in fiber-rich areas, causing out-of-plane undulations in the fibers being placed. In the micrograph shown in Figure 18, the waviness of fibers in 0-degree ply placed on 90-degree ply is observable around the resin-rich region of 90-degree ply.

**Figure 19.**
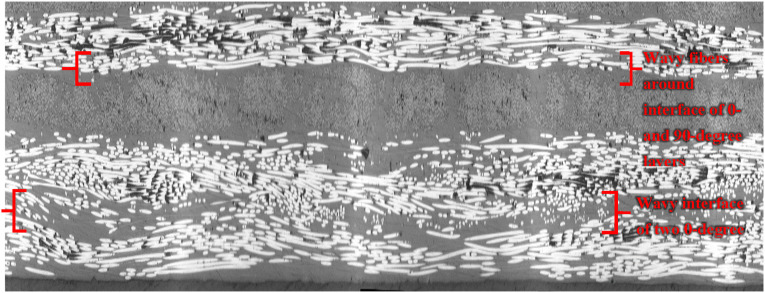
Out-of-plane waviness of fibers caused by non-uniform fiber content.

This waviness could occur at interface of different-angle or same-angle layers. Figure 19 represents another interfacial waviness of two consecutive 90-degree and 0- degree plies and also waviness of two consecutive 0-degree plies. The micrograph is vertically stretched by a factor of 5 in order to clearly depict the waviness. Apparently, the origin of this defect is variation of fiber content across width inside supplied impregnated tape.

##### Missing-tow-induced out-of-plane waviness

**Figure 20.**
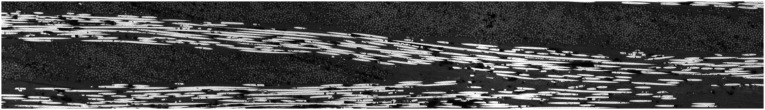
Ply out-of-plane waviness caused by missing tow on previous ply.

Missing tow can be considered as a defect similar to a gap with a width equal to the width of consolidated tape. In Figure 20, a missing tow is discernible on the right side of microscopic image where the top 0-degree ply descends and comes into contact with the previous 0-degree ply.

##### Splice/ply-drop-off -induced out-of-plane waviness

**Figure 21.**
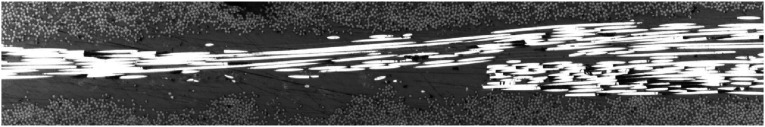
Out-of-plane waviness caused by splice.

The occurrence of a splice may create localized undulations, exhibiting an amplitude equal to half of the consolidated ply thickness. As shown in Figure 21, apart from transverse deflection of the top 0-degree tape, large resin-rich areas become apparent beneath it. The explained defects are also present in ply drop-off. Although a single ply drop-off may induce similar out-of-plane waviness to that of a splice, multiple ply drop-offs create more pronounced waviness that could propagate through several subsequent layers.

##### Intra-layer out-of-plane fiber waviness

**Figure 22.**
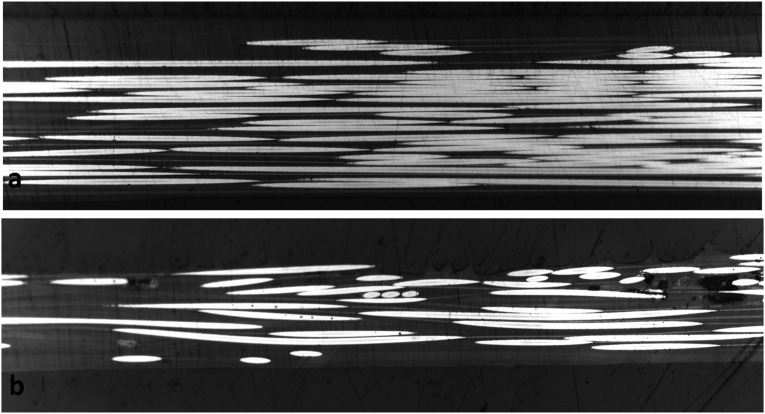
Side-view cross-section (cut-plane along fibers) of (a) unprocessed tape (b) AFP-consolidated tape.

The defects outlined so far in this section elucidated out-of-plane waviness on the level of ply. They could also be considered localized wrinkles of plies. Nevertheless, beyond ply waviness, waviness of fibers within a ply at a smaller scale may also influence the properties of ply. The out-of-plane waviness of fibers even exists in a flat and uniformly thick ply. In the previous section on defects in supplied tapes, the out-of-plane waviness ratio was estimated to be 303. The side-view cross-section of prepreg tape prior to process is shown again in Figure 22(a) and compared with that of a tape processed by AFP (in Figure 22(b)) with HGT temperature of 950°C and compaction force of 80 lbf on a flat steel mandrel.

The increase in fiber angle deviation is an indicator of elevated out-of-plane waviness of fibers. The standard deviation of fiber angles increased from 0.5° in prepreg tape to 1.7° in AFP-processed tape, and consequently, the waviness ratio decreased by 3.4 times from 303 to 90. Essentially, the exertion of concentrated pressure by compaction roller and the complex interactive dynamics of fibers during rapid deformation of composite tape lead to the bending of fibers.

#### In-plane waviness of fibers

**Figure 23.**
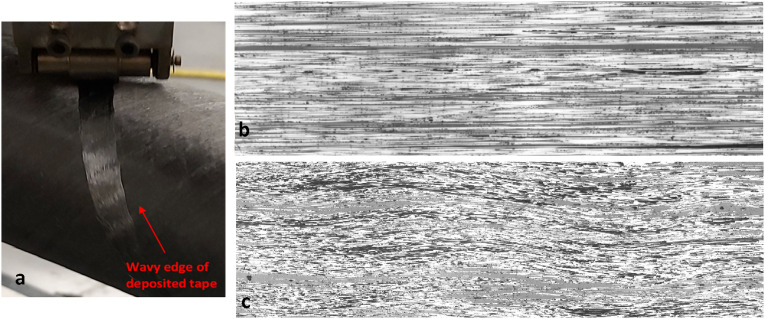
(a) In-plane waviness of deposited tape in manufacturing of composite tube using AFP (Without steering; tape trajectory has no in-plane curvature) – (Right) microscopic top-view (cutting plane parallel to the plane of laminate) for (b) supplied tape before AFP process (c) AFP-consolidated tape within laminate with actual aspect ratio.

The in-plane waviness of fibers has also attracted significant attention, but mostly confined to TSCs. In a relatively recent experimental investigation by Sitohang *et al.*,^73^ the negative influence of in-plane fiber waviness on compressive strength of quasi-isotropic CF/PEEK laminates made by stamp forming appeared to be significant, where waviness triggers initiation of damage primarily via kinking mechanism. In the context of thermoplastic forming processes, including thermoforming or compression molding, the application of non-hydrostatic pressure and transverse flow may result in lateral movement of the fibers and increased in-plane waviness.^74^ Specifically, during the in-situ consolidation process, the compaction roller and substrate constrain and compress the top and bottom surfaces of incoming tape, whereas the side edges of tape are free, culminating in transverse flow of tape and spreading fibers. This dynamic phenomenon, in which fibers are subjected to multiple contact forces from neighboring fibers during rapid deformation of tape, can lead to the increase of in-plane waviness of fibers in each ply. Figure 23(a) depicts the rear side of compaction roller during deposition of a CF/PEEK band on a composite tube. The existence of in-plane waviness in processed tape renders gaps/overlaps impossible to avoid, as wavy adjacent bands cannot fit their edges together.

The in-plane waviness of fibers within a processed tape can be compared against that of unprocessed impregnated tape. During the impregnation process of fibers, the tension on fiber maintains them quite straight. The in-plane waviness ratio of fibers in supplied tape was previously calculated to be 296 using the top-view micrograph (Figure 23(b)). For a layer in the cross-ply laminate made by AFP, the top view of fibers within mid-section is presented in Figure 23(c) with maintained actual aspect ratio. Both micrographs in Figure 23(b) and 23(c) were taken with the same magnification. The measured amplitude and wavelength of waviness in the consolidated layer are 85 µm and 4074 µm, respectively. The resulting waviness ratio is 24, signifying a 12.3-fold reduction in waviness ratio (increase in waviness severity) after the process. Compared with the previous section, the increase in the severity of in-plane fiber waviness is greater than the increase in severity out-of-plane fiber waviness.

**Figure 24.**
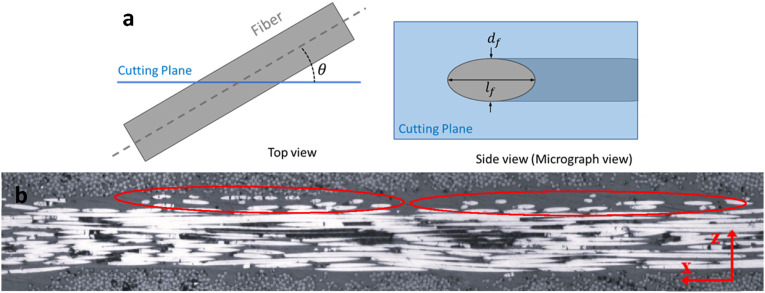
(a) Cross-sectional view of fiber cut by a plane (b) Wandering fibers in topmost portion of layer.

As an alternative method, the in-plane waviness of fibers can also be assessed in side-view micrographs (plane of view perpendicular to plane of laminate but parallel to fibers), where fiber paths are visible throughout the ply thickness. An element of fiber with cylindrical shape, once intersected by a cut plane (microscopic plane of view), appears as an ellipse as shown in Figure 24(a).

**Figure 25.**
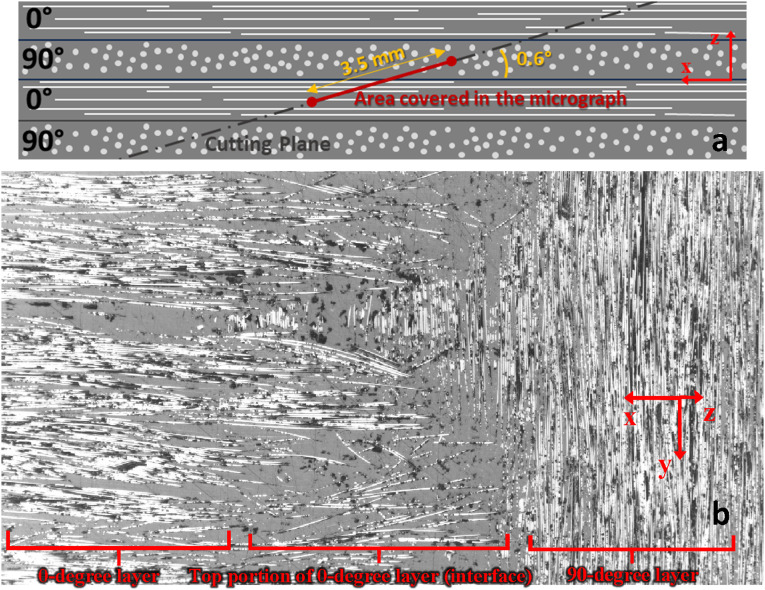
Inclined top-view micrograph of two layers (a) schematic position, orientation, and size of the micrograph (b) the microscopic image.

The local angle of fibers with respect to the cutting plane can be calculated using dimensions of ellipses in micrographs, i.e., $\theta =\sin^{-1}\left(d_{f}/l_{f}\right)$ , where $d_{f}$ is fiber diameter, and $l_{f}$ is visible length of fiber (larger diameter of ellipse). A layer within the same laminate is illustrated in Figure 24(b). Observations propose most of the fibers in the upper part of layers have shorter visible lengths, indicating larger deviation angles, and therefore larger amplitude of in-plane waviness in the upper side of layer, close to the interface with the next layer. This was verified with a slightly inclined top-view micrograph that passes two 0-degree and 90-degree layers in the laminate (Figure 25(a)). The severe in-plane waviness of fibers at interface of layers is evident in Figure 25(b).

The pronounced in-plane waviness in the upper surface of layers may be attributed to the fibers that come in contact with compaction roller during in-situ consolidation. The compaction roller exerts traction on incoming fibers on the top surface of tape, causing them to become disarranged.

#### Cracked fibers

**Figure 26.**
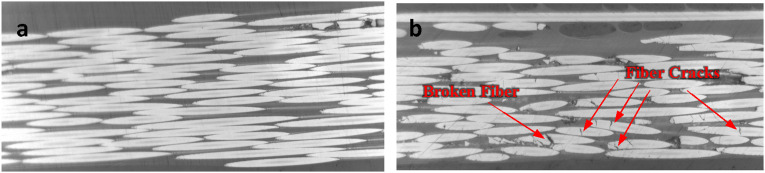
Side view of fibers in (a) unprocessed tape (b) AFP-consolidated tape.

The in-situ nature of AFP offers very short consolidation times, compared with that of autoclave processing or press molding. To attain laminates of similar quality, localized application of larger pressure and higher temperature is implemented on the material to expedite the full intimate contact and void elimination. The magnitude for local pressure exerted via hard roller is on order of hundred bars (few hundred newtons force applied on several square millimeter compaction area), which is significantly greater than typical pressure used for autoclave consolidation (7 bars recommended for Cytec APC-2-PEEK^75^) or press molding (14 bars recommended for Cytec APC-2-PEEK). This equals to application of extreme localized transverse force on fibers of incoming tape. Given the remarkably lower strength of carbon fibers in the radial direction compared with the axial strength, the localized bearing stresses exerted by the neighboring fibers can create or extend cracks in fibers (Figure 26). It should be noted that some cracks can originally exist in input tape (e.g., pac-man cracks running along length) but may grow by application of force. This defect requires elaborate investigation at nano and micro scale, and the effect of cracked fibers on longitudinal and transverse properties of consolidated ply needs to be experimentally assessed.

#### Voids

The prevalence of voids as common defects in polymeric fiber-reinforced composites has attracted vast research works on different aspects of formation, inspection, and impacts. Voids in composite materials are undesirable owing to reductions in matrix dominated properties, including transverse strength and stiffness, as they serve as stress concentration points contributing to structural failure.^76,77^ TPC tapes could exhibit around 5% void volume fraction, whereas void volume fraction of less than 1% is mandated for high performance parts.^78^ In contrast to consolidation of TSCs where voids could migrate out of laminate prior to rapid scalation of cure degree, in the course of in-situ consolidation of thermoplastic tapes the voids could only be compressed and vanished through the application of surrounding pressure, due to brief time frame and high resin viscosity.

Intra-laminar void consolidation is affected by several factors, especially resin pressure, temperature history, and consolidation time. Ranganathan and Advani^78^ developed a void consolidation model that incorporates void growth and transport phenomena to predict intra-laminar void fraction. It has been demonstrated that at outside consolidation region void growth may take place and increase final void fraction.^79^ Although the void fraction may even drop to the order of 0.1% under compaction roller, the voids may grow after release point due to their elevated internal pressure and low modulus of matrix in temperatures above Tg. Hence, forced convective cooling on the rear side of roller or adding cold rollers have been implemented to curb void growth.^79^ While deconsolidation of processed tape has been explained by elastic rebound of compressed voids, the effect of pull-off force exerted by roller on tape at release point is yet to be investigated and associated with deconsolidation.

Inter-laminar voids, nevertheless, rely on the development of intimate contact, and generally appear if the surface of incoming tape and substrate fail to achieve full contact. The establishment of full intimate contact is significantly influenced by surface roughness^80^ and uniformity of widthwise thickness in supplied impregnated tapes, considering limited time frame of consolidation. Additionally, uneven and fluffy surface profile of substrate as a result of pull-off force between metallic roller and thermoplastic resin will be shown in another section, in which surface asperities and pulled-off fiber bundles had dimensions in the same order of magnitude with tape thickness. This process-induced surface roughness may potentially increase required time or pressure for attaining full intimate contact in the course of placing a subsequent layer. Upon full intimate contact, the inter-laminar voids might still reappear if interfacial temperature exceeds Tg after compaction region.

#### Incomplete healing

Once the molten surfaces of incoming tape and substrate make contact at nip point, the interdiffusion of the polymeric chains across the interface commences, commonly referred to as healing or autohesion. Full interlayer healing is contingent upon establishing full intimate contact. Butler *et al.*^81^ conducted an examination for the ratio of time scale for full healing to that of intimate contact. Their analysis revealed that in context of tow placement, both mechanisms are controlling. To attain full healing after full intimate contact, the interfacial migration of polymer chains must continue until the interface turns into a cohesive matrix material, thereby achieving maximum bond strength. The time required for complete interfacial welding of thermoplastic polymer at isothermal conditions can be presented as^82^:

(10) $$t_{w}=A\;\exp\left[\frac{E_{a}}{R}\left(\frac{1}{T}-\frac{1}{T_{ref}}\right)\right]$$

where A = 0.11 sec, $E_{a}$ = 57.3 kJ/mol, R = 8.314 kJ/kmol.K, and $T_{ref}=$ 400 °C for AS4/PEEK 150.^83,79^ Typical processing temperatures for the material fall within the range of 370 °C–430°C, which results in a required time for full healing between 71 and 177 milliseconds. While this time frame is significantly shorter than the time scale associated with autoclave consolidation or compression molding, it may provide a critical role in the context of in-situ consolidation. As a temperature-dependent phenomenon, autohesion can continue to develop even when the compaction roller moves away. However, for optimized AFP equipment under normal process parameters, interface temperature is above melting point only before arriving at release point. The range of layup velocity can lie within 20 mm/s to 200 mm/s in typical in-situ CF/PEEK consolidation practices^23^ and contact length for the hard roller is on the order of 2 mm.^80^ Hence, consolidation time may vary from 10 to 100 milliseconds. Even if applied compaction pressure is large enough to render intimate contact time fairly small and maximize available time for healing, a comparison of above-mentioned time scopes highlights the potential for incomplete healing in the context of high-speed AFP deposition. Elevating nip point temperature may expedite the onset of healing and lower the required time for complete healing. Nonetheless, increasing temperature to allow high-speed deposition encounters the concern of material degradation before nip point.

#### Matrix degradation

Qureshi *et al.*^26^ indicated the thermal degradation of thermoplastic matrix as a factor contributing to the reduction of interlaminar shear strength in automated tape placement. The importance of thermal degradation lies in the potential for causing irreversible damage. The thermal degradation of PEEK leading to mass loss falls within the temperature range of 575°C–580°C,^84^ whereas the initial reactions of degradation, namely chain scission and cross-linking start at lower temperatures, encompassing in-situ consolidation process window.^85^ Onsets of cross-linking and chain scission for PEEK have been remarked by prolonged exposure to temperatures above 400°C^86^ and 450°C,^87^ respectively. The final degradation degree in processed material is influenced not solely by temperature, but also the exposure time to that temperature. The exposure time is directly related to material deposition speed. Pitchumani *et al.*^88^ noted that increase in deposition speed leads to a reduction in the exposure time to heat, resulting in a reduction in the degradation degree. In that parametric analysis, the heat input by torches and rollers was kept constant and deposition speed was varied. However, a general requirement for melting material up to suitable processing temperatures calls for increased heating power input when higher deposition speeds are sought. For a given nip point temperature in course of torch-assisted AFP equipment, increasing deposition velocity brings about escalated through-thickness temperature gradient on incoming tape. In rapid depositions, given the relatively low transverse thermal diffusivity of CF/PEEK, the temperature of torch-exposed surface on incoming tape could exceed degradation temperature, while the rear side of tape is yet to even reach Tm. Even if overheated portion of thickness is small, degraded resin on surface of incoming tape impairs interlaminar healing.

**Figure 27.**
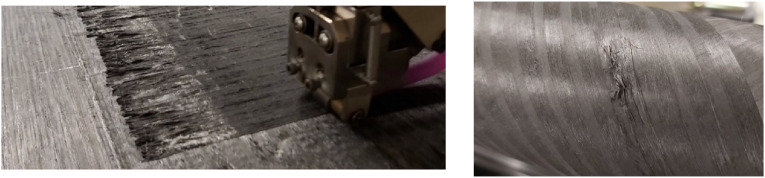
(a) Overheated material at the start of the bands. (b) Decomposed tape at splice due to overheating.

In HGT-assisted AFP systems, there is also an increased risk of degradation at the starting point of bands. This is due to the duration it takes for the placement head to reach its nominal velocity from zero, prolonging exposure of tape to the heating source. Furthermore, the excessively hot roller after the off-part travel of head can burn the tape at the outset of band. Figure 27(a) exhibits a laminate being manufactured under standard process parameters, where the beginnings of the bands are distorted due to overheating. In AFP systems using other heating systems that can offer fast response time and high controllability (e.g., diode lasers) the chance of degradation can be minimized for such conditions.

#### Steering-induced defects

**Figure 28.**
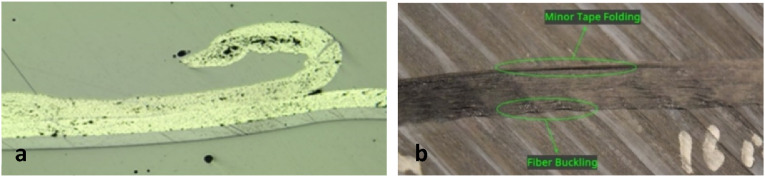
(a) Fiber pull-up at the outer edge^66^ (b) Fiber buckling and tape folding in steered sample^93^.

In the previous sections of this paper, various defects were discussed that may generally occur in thermoplastic laminates made by AFP, either flat or curved parts. However, in more complex AFP practices when tape is steered along non-geodesic path, additional defects may arise. By steering thermoset prepregs, several defects namely wrinkle and blister (out-of-plane buckling of fibers), steering-induced waviness (in-plane buckling of fibers), fold or tow pull up, and sheared fibers have been studied by various authors.^89–92^ Prepreg tack, influencing the interaction of deposited tape and substrate, is one of the major parameters affecting the formation and development of these defects. In the course of in-situ consolidation of TPCs, however, the solidification of interfacial resin influences the adhesion of tape to substrate and development of steering-induced defects. On top of that, the viscoelastic properties of deposited thermoplastic tape vary over a wide range from temperatures over Tm to sub Tg, increasing the complexity of steering-induced defects. Final defects in AFP-steered thermoplastic tapes have been identified experimentally in few works in recent years.^66,93^ Clancy *et al.*^66^ studied the effect of deposition speed and steering radius on geometrical dimensions and bond strength of manufactured samples. It has been concluded that high deposition speed may lead to fiber pull-up (Figure 28(a)), attributed to inadequate consolidation. Rajasekaran and Shadmehri^93^ explored the effect of steering radius, deposition speed, substrate angle, and repass on geometrical attributes and bond strength of the samples using observational analysis and lap shear testing, respectively. The defects observed below a critical steering radius were fiber buckling at the inner edge due to compression and tape folding on outer edge due to tension, shown in Figure 28(b).

### Macro-scale defects

#### Splice

The presence of splice introduces an area of discontinuity in fibers and a potential site for stress concentration. But from a manufacturing point of view, splice as an end point of one band and start point of the next band, is a point of discontinuity in operation of AFP equipment on which temperature distribution in incoming tape could become unsteady or uncontrollable. For an HGT-assisted AFP head, the incoming tape could be overheated at start point of each band. During the off-part travel or positioning of AFP head, compaction roller is directly exposed to hot gas torch reaching to its maximum temperature. At start point of band, the matrix material could be overheated and degraded once incoming tape touches the roller. Moreover, for high deposition speeds, a certain time is required for the head to reach its designated velocity, increasing absorbed heat by tape at the beginning of band. In Figure 27(b), a worn-out band due to matrix decomposition at splice area is illustrated. Not only does burnt matrix disable load-bearing capacity of tape, but also it acts as a foreign object debris and impairs local adhesion of the subsequent layer to substrate. Splice creates more secondary defects, including resin-rich pockets and out-of-plane waviness as was elaborated upon previously.

#### Missing tape or fiber bundle

**Figure 29.**
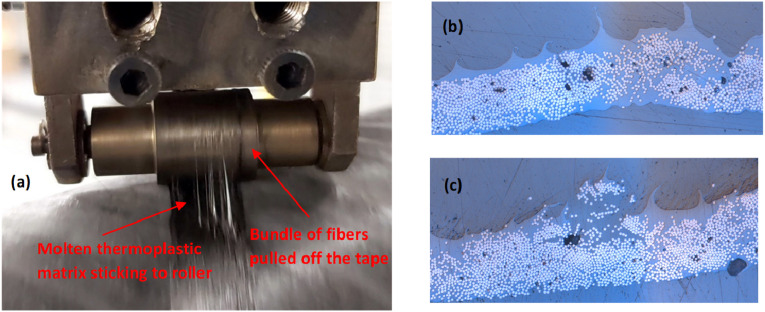
(a) Thermoplastics/fiber-bundles sticking to roller after consolidation: causing (b) wavy surface profile on processed tape (c) detached fiber bundles on top surface.

The incident when a band or a significant portion of band is missed within a ply is considered an important defect that can potentially create a site for progressive delamination failure.^8^ Missing tape can happen for three main reasons - placement flaw, finished material on spool, or tape sticking to compaction roller instead of being welded to substrate. While the first two reasons were previously explained, missing tape could also occur if incoming tape adheres to compaction roller and wraps over it throughout the band. This happens when tape-roller adhesion is stronger than tape-substrate bond, specifically when tape temperature at release point is close to (or larger than) melting point of thermoplastic matrix. Molten thermoplastic polymer can wet the surface of compaction roller and spread out over surface. The wetting is pronounced for the case of metallic compaction roller whose surface energy is significantly greater than that of polymers. By surface wetting, Van der Waals attractive forces increase between interfacial molecules of the tape (resin-rich area on top surface) and the roller. Furthermore, micro-roughness on surface of compaction roller increases surface wettability^94^ and also leads to mechanical interlocking due to infiltration of polymeric molecules into micro-pores of roller surface. The rear side of compaction roller during deposition is shown in Figure 29(a), depicting the tendency of tape for adhesion to roller. Surface profile of a processed tape was also evaluated via micrography as shown in Figures 29(b) and 29(c), highlighting locally protruded areas (resembling ocean waves) and locally pulled-off fiber bundles by roller, respectively. The increased surface irregularities on the top surface of processed tape (Figures 29(b) and 29(c)) increase required time for full intimate contact during deposition of next layer. Furthermore, the negative pressure on tape at release point potentially facilitates deconsolidation and recovery of voids.

Although the adhesion of fiber bundles (a small portion of tape) to roller is a frequently observed defect, missing tape (adhesion of entire tape to roller) occasionally occurs at start point of band.

#### Bridging

**Figure 30.**
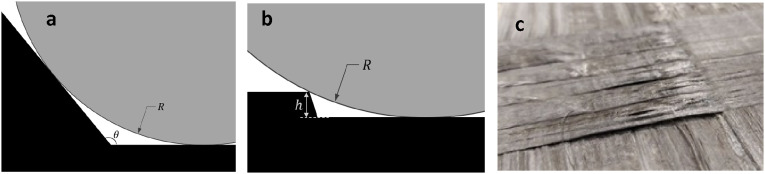
Hard compaction roller passing a (a) concave corner (b) step, (c) Bridging of thermoplastic tapes after passing a step.

Bridging of fibers happens at concave areas where tension on tape overrides its adhesion to substrate, forcing the tape to lift off the part to form a straight shortcut between two points. In AFP of TPCs, tape bridging can take place where deposition path traverses a concave corner (Figure 30(a)) or a step (Figure 30(b)). As depicted in Figure 30(c), bridging of thermoplastic composite tapes after passing a step can be observed. One example of a step is areas with multiple ply drop-off.

If temperature of incoming tape at release point (point of separation between incoming tape and roller) is less than the glass transition temperature (Tg), then the bridging could be minimized due to the formation of solid and strong bond between tape and substrate. However, if release point temperature is larger than heat deflection temperature of material, high tension on tape can magnify bridging length. In cases involving hard compaction rollers, the bridging may become inevitable at concave corners or steps. The minimum bridging length of tape for a concave corner and a step can be represented by the following relations, respectively:

(11) $$l_{c_{\min}}=R\left(\pi -\theta \right)$$

(12) $$l_{s_{\min}}=R\;\cos^{-1}\left(1-\frac{h}{R}\right)$$

where R , θ , and h are compaction roller diameter, corner angle, and step height, respectively. The bridged tape is also deprived of consolidation due to lack of contact with substrate for compaction at deposition, resulting in elevated voids. In automated manufacturing of TSCs, the secondary process of autoclave curing may attenuate gap area between bridged tapes and corner by pushing fibers toward corner under autoclave pressure (also can be done by vacuum debulking^8^) or by filling the corner gap with flow of excess resin. But in context of AFP of TPCs as an out-of-autoclave process, the defects are final after the deposition of the last layer.

#### Warpage

One of the major issues with open-section thermoplastic structures manufactured by automated fiber placement is the warpage, which often appears when the part is removed from the mold. High processing temperature of thermoplastic composites produces significant residual stresses relative to their strength.^95^ The formations of residual stresses in thermoplastic composites are classified at three different micro-mechanical, macro-mechanical, and global levels,^96,97^ where the latter two are mainly responsible for out-of-plane distortion.

**Figure 31.**
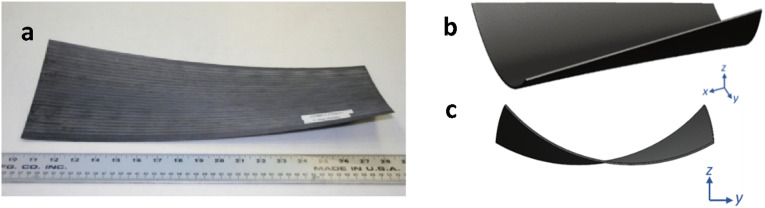
(a) Distorted unidirectional TPC made by AFP^56^ (b) isometric view (c) front view.

Global manufacturing-induced stresses are generated by uneven development of stresses due to spatially non-uniform solidification and shrinkage of thermoplastics. Steep through-thickness thermal gradients as a result of rapid cooling are accountable for significant global stresses. In AFP of TPCs, the in-situ placement process inherently involves concentrated through-thickness and in-plane thermal gradients around the deposition area. The outcome will be the unsymmetrical and uneven stresses formed through thickness which contribute to warpage. In this context, a unidirectional laminate manufactured using AFP is shown in Figure 31(a). A general geometry of unidirectional thermoplastic laminates made by AFP is depicted in Figures 31(b) and (c) using isometric and front views, in which material deposition direction was along the x-axis. The major characteristic is combined bending curvature ( $\kappa_{y}$ ) and twist ( $\kappa_{yx}$ ) in the final shape.

During accumulative in-situ placement of tapes on laminate, the temperature of incoming tape and substrate (previously deposited layer) exceed melting point, experiencing stress-free condition. Stress relaxation may also occur for some of the top layers that exceed Tg, depending on their temperature and dwell time. When roller moves away, the stress-free layers on top start to cool down and shrink transversely, while the other layers are relatively cold and stiff. This interaction develops tensile stress on a top layer and exerts compressive stress on the bottom ones. Consequently, by detachment of part from mold, the upward curvature comes into view. The combination of crystallization shrinkage and thermal contraction results in the development of residual stresses,^98^ during which elastic modulus is also simultaneously changing.^99^ The stress relaxation effect could also alter final residual stresses^98,100^ and severity of warpage. Crystallization temperature, corresponding volumetric shrinkage, and final degree of crystallinity are strongly dependent on local cooling rate history of semi-crystalline thermoplastic material.^99^ Furthermore, the degree of crystallinity also governs final modulus of material,^101^ which determines compliance of plies to developed stresses.

To counteract this defect, residual stresses can be relaxed by a heated mandrel to retain all layers above the Tg throughout the process.^102^ This procedure, however, could increase the likelihood of deconsolidation and elevation of interlaminar voids. If a heated mandrel proves to be impractical or economically unfeasible for large-scale structures, reducing the deposition speed may assist with mitigation of residual stresses.^103^ At lower speeds, more layers reach high temperatures,^104^ and deeper stress relaxation along with more uniform temperature distribution decreases final curvatures.

## Summary

The summary of potential defects appearing in in-situ consolidated thermoplastic composites made by automated fiber placement along with their reasons and potential effects can be listed as follows:

**Table:** 

Name and Illustration	Reasons	Potential Effects
Gaps and overlaps 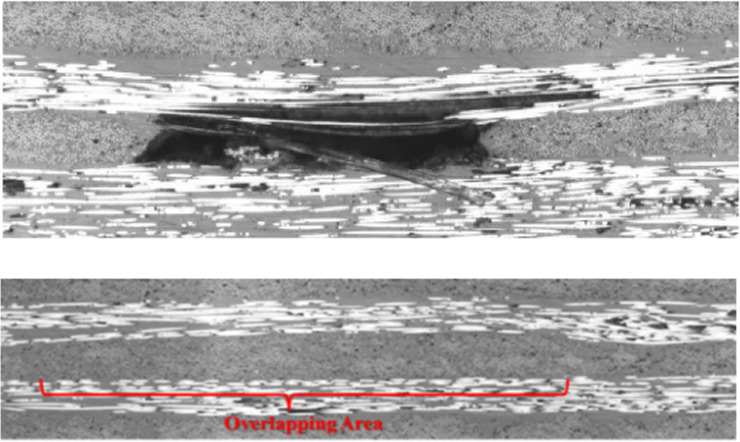	• AFP equipment positioning errors	• Introducing holes/voids at gap area
	• Sensitivity of consolidated tape width to process parameters	• Causing out-of-plane waviness in subsequent plies
	• Variation in local width of consolidated tape after non-uniform transverse squeeze flow along length	
	• Variation in local width of processed tape after fiber steering	
Out-of-plane waviness 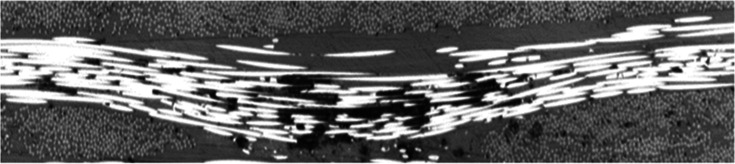	• Gaps and/or overlaps in a previous ply • Soft resin-pockets in a previous ply • Missing tape in a previous ply • Splice/Ply-drop-off in a previous ply • Intra-ply fiber waviness due to dynamics and interaction of neighboring fibers during rapid consolidation	• Creating local out-of-plane normal stresses and interlaminar shear stresses • Initiating interlaminar shear failure and delamination
		• Diminishing Young’'s modulus and compressive strength
In-plane waviness 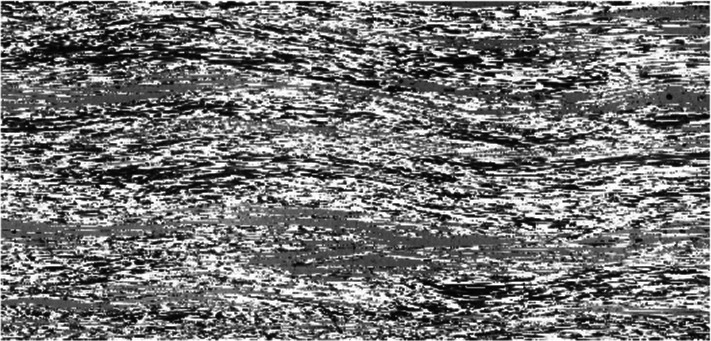	• Transverse migration and bending of fibers due to localized pressure of roller, while insufficient tension on fibers is applied	
	• Variation in width of processed tape under non-uniform transverse squeeze flow	
Cracked fibers 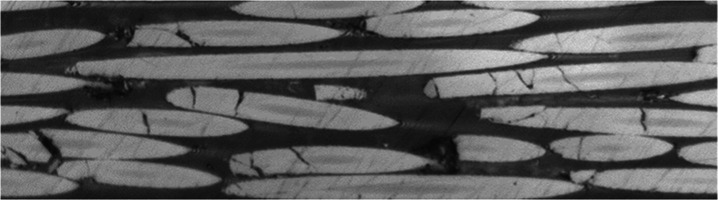	• Fiber cracks in supplied impregnated tapes	• Potential reduction in transverse strength of composite ply due to longitudinal orientation of cracks
	• Excessive lateral compressive stress on fibers during in-situ consolidation via hard roller	
Voids 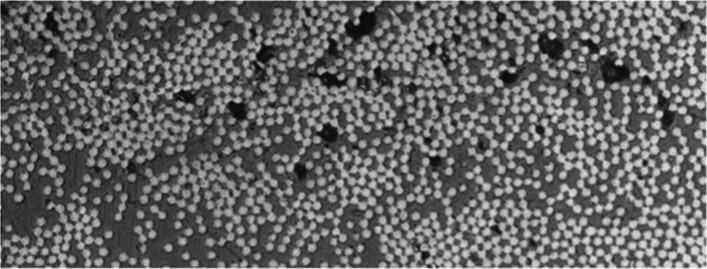	• Intralaminar voids originating from voids in supplied impregnated tapes	• Stress concentration points contributing to structural failure
	• Interlaminar voids caused by incomplete intimate contact	• Reductions in matrix dominated properties, including transverse strength
	• Deconsolidation after the compaction roller	• Interlaminar voids expediting onset of delamination
Incomplete healing 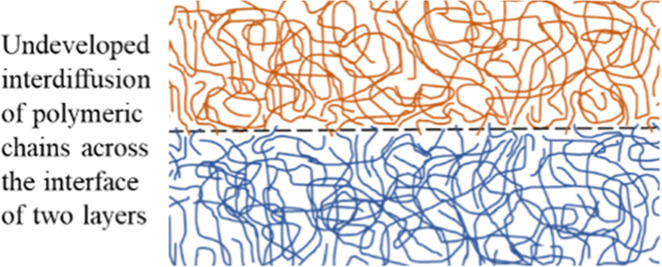	• Insufficient time above melt temperature at interface of incoming tape and substrate	• Weak interlaminar bonding
Matrix degradation 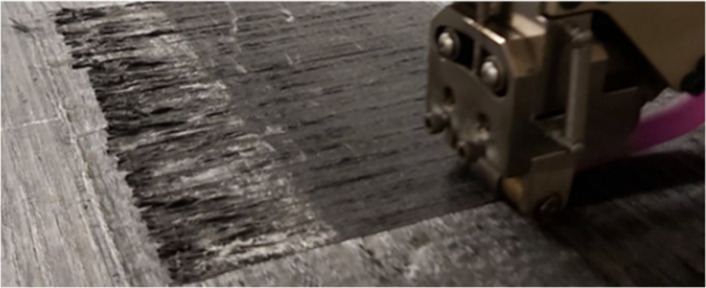	• Over-exposure of thermoplastic matrix to heat source	• Decreased transverse strength of ply
		• Reduction of interlaminar shear strength
Fiber pull-up or folding/Fiber buckling in steering 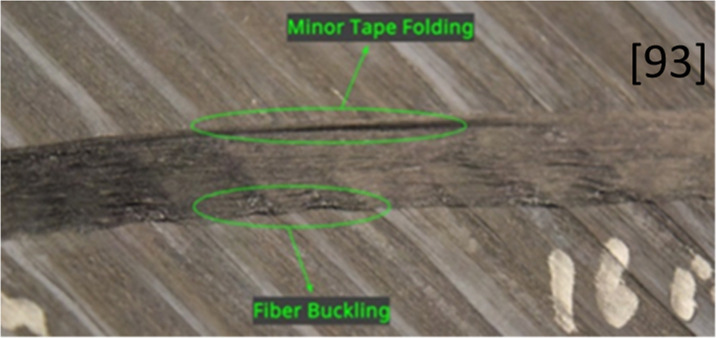	• Folding; tension on outer edge during fiber steering	• Reduction in lap shear strength for tight radii of steering
	• Fiber buckling; compression on inner edge during fiber steering	
Splice 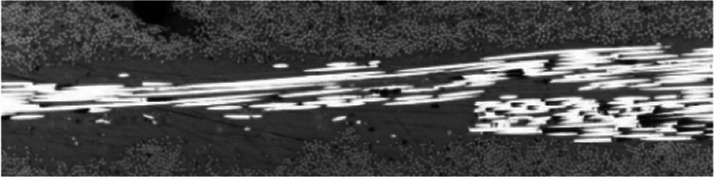	• Splices in supplied impregnated tape spools	• Causing out-of-plane waviness in subsequent plies
	• Finished spool at mid-band of fiber path	• Elevated possibility of material degradation
Missing tape or fiber bundle 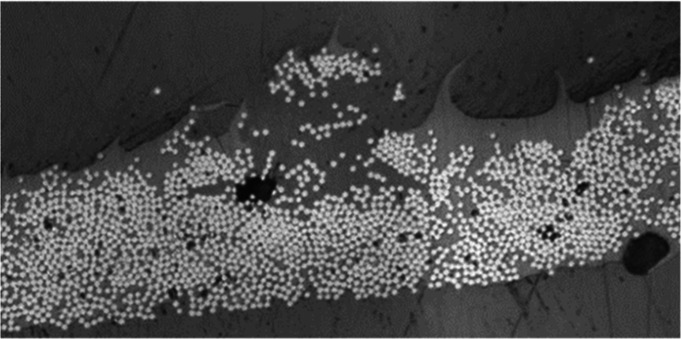	• Placement head fault	• Missing tape causing out-of-plane waviness in several subsequent plies
	• Tape or fiber bundle sticking to and wrapping around the roller	
Bridging 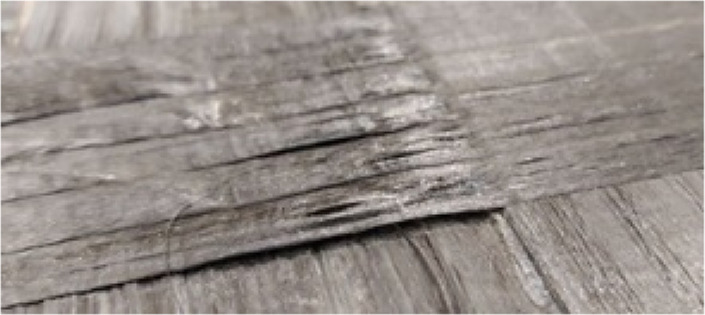	• Concave corners on tool	• Preventing consolidation of incoming tape
	• Steps due to multiple ply drop-off	• Introducing holes/voids
		• Deviation from intended shape at edge
Warpage 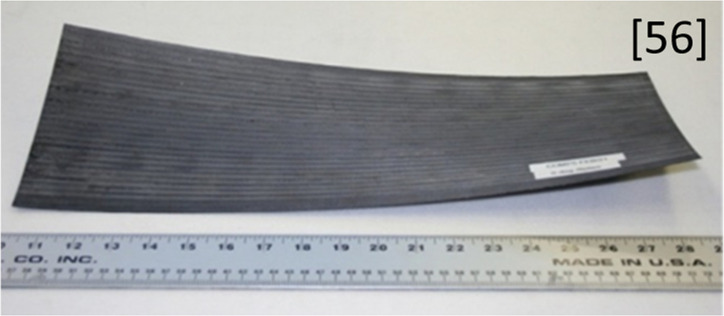	• Global manufacturing-induced residual stresses; non-uniform cooling and subsequent formation of unsymmetric stresses across thickness in the laminate	• Deviation from design geometry
		• Adverse effect on structural stability

## Concluding remarks

In this study, the first step aimed to identify imperfections in thermoplastic impregnated tapes used for AFP. Following that, a succinct elucidation of prevalent defects and limitations in performance of AFP equipment was presented, acting as origins of certain defects. Subsequently, final micro-scale defects (e.g., overlaps and gaps, in-plane and out-of-plane waviness, fiber cracks, voids, incomplete healing, matrix degradation, and steering-induced fiber pull-up or buckling) and macro-scale defects (e.g., missing tape, splice, bridging, and warpage) were characterized. This paper aimed to portray a clear picture of various defects in TPCs made by AFP, based on observation of CF/PEEK tapes in-situ consolidated using HGT heating system and hard roller. For the case of some defects that were already studied extensively in the past (e.g., voids) the description is limited to a succinct review, while the ones with limited understanding (e.g., in-plane and out-of-plane waviness) were supported by our observation and examination. Certain defects presented in this work (e.g., in-plane and out-of-plane waviness, voids, and warpage) may appear in other manufacturing methods such as compression molding or autoclave consolidation, while the rest can be specific to AFP. However, given the clear distinctions between AFP and conventional processes from different aspects, even the defects widely investigated in conventional processes require separate and focused studies in the context of AFP of TPCs. It should be noted that while the presented list aimed to discuss the major defects that are usually undesirable in the final part, additional parameters can still influence the final properties and quality of the composite part but may not be considered a defect. For instance, crystallinity as a state of the matrix material is inversely correlated with cooling rates. Faster cooling rates observed in AFP compared to that of autoclave processing may result in lower stiffness^105^ and strength,^106^ but higher fracture toughness^35^ an higher resistance to impact and delamination.^107^ Depending on the application, the process parameters and AFP configuration need to be tailored to satisfy specific structural requirements. Lastly, this study in defect classification and characterization can be followed by elaborate experimental studies on the impact of defects on the performance of TPCs.
